# Biomechanics of Forearm Rotation: Force and Efficiency of Pronator Teres

**DOI:** 10.1371/journal.pone.0090319

**Published:** 2014-02-28

**Authors:** Pere Ibáñez-Gimeno, Ignasi Galtés, Xavier Jordana, Assumpció Malgosa, Joan Manyosa

**Affiliations:** 1 Unitat d'Antropologia Biològica, Departament de Biologia Animal, Biologia Vegetal i Ecologia, Universitat Autònoma de Barcelona, Bellaterra, Barcelona, Catalonia, Spain; 2 Centre de Patologia Forense de Collserola, Institut de Medicina Legal de Catalunya, Montcada i Reixach, Barcelona, Catalonia, Spain; 3 Unitat de Medicina Legal i Forense, Departament de Psiquiatria i de Medicina Legal, Universitat Autònoma de Barcelona, Bellaterra, Barcelona, Catalonia, Spain; 4 Departament de Paleobiologia, Institut Català de Paleontologia Miquel Crusafont (ICP), Universitat Autònoma de Barcelona, Bellaterra, Barcelona, Catalonia, Spain; 5 Unitat de Biofísica, Departament de Bioquímica i de Biologia Molecular, and Centre d'Estudis en Biofísica, Universitat Autònoma de Barcelona, Bellaterra, Barcelona, Catalonia, Spain; University of Utah, United States of America

## Abstract

Biomechanical models are useful to assess the effect of muscular forces on bone structure. Using skeletal remains, we analyze pronator teres rotational efficiency and its force components throughout the entire flexion-extension and pronation-supination ranges by means of a new biomechanical model and 3D imaging techniques, and we explore the relationship between these parameters and skeletal structure. The results show that maximal efficiency is the highest in full elbow flexion and is close to forearm neutral position for each elbow angle. The vertical component of pronator teres force is the highest among all components and is greater in pronation and elbow extension. The radial component becomes negative in pronation and reaches lower values as the elbow flexes. Both components could enhance radial curvature, especially in pronation. The model also enables to calculate efficiency and force components simulating changes in osteometric parameters. An increase of radial curvature improves efficiency and displaces the position where the radial component becomes negative towards the end of pronation. A more proximal location of pronator teres radial enthesis and a larger humeral medial epicondyle increase efficiency and displace the position where this component becomes negative towards forearm neutral position, which enhances radial curvature. Efficiency is also affected by medial epicondylar orientation and carrying angle. Moreover, reaching an object and bringing it close to the face in a close-to-neutral position improve efficiency and entail an equilibrium between the forces affecting the elbow joint stability. When the upper-limb skeleton is used in positions of low efficiency, implying unbalanced force components, it undergoes plastic changes, which improve these parameters. These findings are useful for studies on ergonomics and orthopaedics, and the model could also be applied to fossil primates in order to infer their locomotor form. Moreover, activity patterns in human ancient populations could be deduced from parameters reported here.

## Introduction

The effect of muscular forces on bone structure can be predicted from osteometric parameters using biomechanical models if the line of action and the origins of the tendon are known [1], [2]. The rotational efficiency (E_rot_) of the pronator teres (PT) is a measure of its rotational capacity as a function of the applied force [3]. The PT E_rot_ can be calculated from several osteometric parameters of the upper-limb skeleton, basically related to the curvature of the diaphysis of the radius bone, the size and shape of the humeral medial epicondyle, and the proximo-distal location of PT radial enthesis [3]–[5].

During resisted pronation, PT acts as the primary agonist, displaying relatively higher activity levels than pronator quadratus [6]. In contrast to the latter, PT is largely affected by elbow and forearm position [6], [7]. The positioning of the forearm may also influence the components of PT force, which in turn can entail structural changes with different functional implications. Therefore, it can be hypothesized that each component influences the skeletal structure in a different way, e.g. a component may enhance the curvature of the radius when it exerts a bending loading on its shaft [3]. However, the biomechanical behavior and structural effects of these components in the pronation-supination and flexion-extension ranges have never been examined.

This study aims to analyze PT E_rot_ for a full range of elbow flexion angles in order to (i) analyze the relationship between the components of the force vector and the skeletal structure, (ii) assess how E_rot_ is modified by changes in several osteometric parameters and (iii) get further knowledge about the functional implications of the variation of this parameter in the flexion-extension range. The results of this biomechanical analysis will lay the foundations for future studies about comparative and evolutionary anatomy, as well as works on applied sciences, such as ergonomics, sports medicine and orthopaedics, providing relevant information about the relationship between the skeletal structure and PT functionality.

## Materials and Methods

### Materials

The skeletal remains used in this study are part of an osteological collection housed at Unitat d'Antropologia Biològica (Universitat Autònoma de Barcelona). The skeletons in this collection were ceded to Unitat d'Antropologia Biològica (Universitat Autònoma de Barcelona) by Cementiri de Granollers (Ajuntament de Granollers). This cession was subjected to prior agreement between both institutions. Moreover, this study is part of a project (CGL2008-00800/BOS), which was approved by Ministerio de Ciencia e Innovación (Gobierno de España).

Rotational efficiency was calculated for the right upper-limb skeleton (humerus, radius and ulna) of a 32-year-old male from the abovementioned collection. The E_rot_ of the studied individual was previously shown to be within to the human pattern of variation, calculated for full elbow extension (180°) and intermediate flexion (90°) [5]. The biomechanical model used in the current study enables to calculate E_rot_ as function of the elbow flexion angle by using 3D imaging techniques. NextEngine's 3D Scanner was used to obtain a three-dimensional image of the humerus, which was processed with ScanStudio HD software [8]. The image was exported to the modeling software Rhinoceros 4.0 SR1 [9], where planes and axes were defined and measurements were taken.

### Calculation of pronator teres rotational efficiency

**Figure 1:**
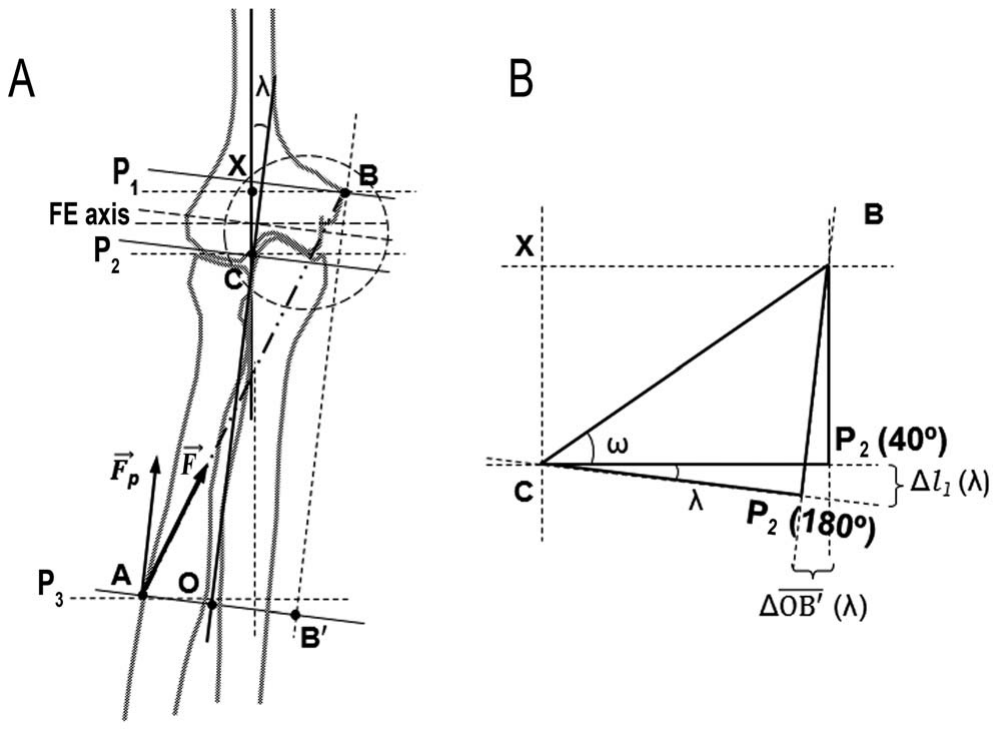
Representation of the influence of carrying angle (λ) on several parameters used to calculate rotational efficiency. **A:** Anterior view of right distal arm and forearm bones in supination position. Humeral and forearm axes, the flexion-extension axis (FE axis), as well as the force exerted by pronator teres muscle (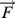, from point A to point B), are represented. 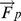 is the vertical component of pronator teres force. Point A indicates pronator teres distal enthesis just at the apex of radial curvature. Point B indicates its proximal attachment site, just at the apex of the medial epicondyle, and point B′ is the projection of point B on plane P_3_. Planes P_1_, P_2_ and P_3_ are parallel to each other and perpendicular to the forearm axis. P_1_ passes through point B, P_2_ passes through the most proximal point of the radial head and P_3_ passes through point A. Point X is the intersection between humeral axis and a plane perpendicular to the humeral axis that passes through point B. Point C is the most distal humeral point of the humeral axis. Point O is the intersection between plane P_3_ and the forearm axis. The dashed circle indicates the zoomed-in area of the right image. **B:** Detail of the left image. Plane P_2_ is represented for a position of full elbow extension (P_2_ (180°)) and for a position of full elbow flexion (P_2_ (40°)). The variation of this plane is caused by the variation of the carrying angle (λ). This causes a variation in *l_1_* and in 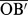 (Δ*l_1_* (λ) and Δ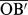 (λ)), which depends on the elbow angle. Angle ω is the angle between plane P_2_ (40°) and 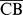 segment.

Galtés et al. [3] developed the biomechanical model to calculate the rotational efficiency (E_rot_) of the pronator teres (PT), which was initially based on the use of CT scan images of the upper-limb. In accordance with this work, E_rot_ is defined by the expression: 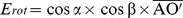



The biomechanical model was later adapted to calculate E_rot_ using skeletal remains by means of photographs of the distal epiphysis of humerus [4]. Although this methodology was simple and practical, the technique was limited since the spatial representation of the humeral planes was not possible. Therefore, some assumptions about the arm and forearm mechanics and the representation of the geometrical points used to calculate E_rot_ had to be made. The use of 3D imaging, basically to define the humeral planes and axes and to determine important details of the medial epicondyle, enabled to obtain more accurate calculations of E_rot_ [5]. As a matter of fact, the calculation of E_rot_ at any elbow position described in the current paper is based on the approach developed by Galtés et al. [4] and Ibáñez-Gimeno et al. [5]. In order to calculate angles α and β at a given forearm and elbow position, several parameters are required (Table 1) [5]. Two of these parameters, ***l*** **_1_** distance and 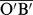 distance, depend on the value of carrying angle (λ) and thus vary throughout the flexion-extension range (Fig. 1) [10]. The carrying angle is the angle between the arm and the forearm long axes (Fig. 1). Assuming a linear variation of this angle between a maximum value (λ_m_) in full elbow extension (180°), which is measured on dry bones [11], and λ = 0° in full elbow flexion (40°) [10], [12]–[14], and using 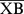 and 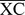 values (Fig. 1B) obtained from the three-dimensional image of the humerus, we can obtain: 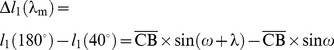
(Eq. 1)

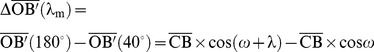
(Eq. 2)


**Table 1:** Osteometric and geometric parameters used to calculate forearm rotational efficiency.

Parameter	Value	Definition
*d_r_*	1.10	Radial head radius
*d_c_*	1.10	Ulnar distal epiphysis radius
*l_f_*	23.00	Physiological length of the radius
*l_pr_*	9.70	Distance between P_2_ and P_3_ planes
λ_180_	10.00°	Carrying angle in full elbow extension
ε	45.65°	Angle between the positive *x*-axis and the position vector of point B
*d_e_*	0.75	Distance between flexion-extension axis and point B
*R_c_*	1.14	Humeral capitulum radius
B	(0.52, −0.53)	Coordinates of point B
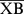	3.93	Distance between point B and humeral axis
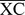	1.34	Distance between P_1_ and P_2_ planes
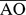	2.60	Curvature of the radius

**Figure 2:**
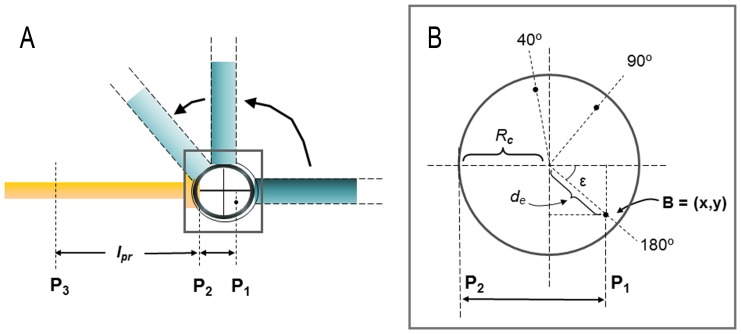
Representation of the changes in position of point B as a function of the elbow angle. **A:** Representation of the elbow in full extension (180°), an intermediate position (90°) and full flexion (40°) from a lateral view. A static radius (yellow) is represented with the humerus in the three positions (blue). Planes P_1_, P_2_ and P_3_ are represented. The point (point B), and so plane P_1_, are shown for 180° of elbow extension. Distance *l_pr_* is the distance between planes P_2_ and P_3_. The distance between planes P_1_ and P_2_ depends on the elbow position. The square indicates the zoomed-in area of the right image. **B:** Change in the position of point B and in the distance 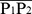 as a function of the elbow angle. The position of point B for the three elbow angles is represented. Point B is positioned in a coordinates system (*x*, *y*) which center is the flexion-extension axis from a lateral point of view. *R_c_* is the radius of the humeral condyle. *d_e_* is the distance between the center of coordinates and point B. Angle ε is the angle between the positive *x*-axis and the position vector of point B.

**Figure 3:**
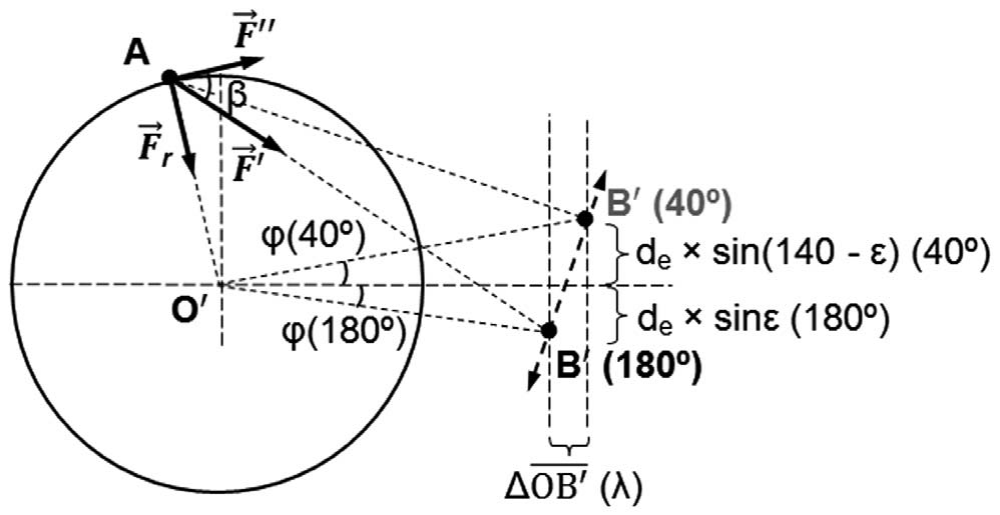
Diagram of plane P_3_ with the main parameters to calculate rotational efficiency. Two elbow positions (180° and 40°) are represented. 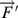 is the projection of the pronator teres force on plane P_3_. 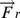 is the radial component of 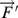. 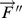 is the component of 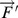 tangential to the rotational movement. Angle β is the angle between 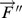 and 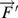. Point A is the radial attachment site of pronator teres. Point O′ is the center of the rotational movement. Point B′ is the projection of point B on plane P_3_. The position of this point varies as a function of the carrying angle of the elbow (Δ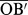 (λ), see Fig. S1) and it is assessed with regards to the horizontal axis by 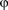 angle. The displacement of point B′ from the horizontal axis can be calculated from the variation of point B position as a function of the elbow angle (see Fig. 2).

Distance values are in centimeters. See Materials and Methods and Figures 1, 2 and 3 for further information about the parameters definition.

Distance *l_1_* (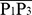) is the sum of 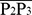 and 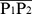 (Figs. 1A and 2A). 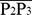 can be measured directly on the radius bone (Figs. 1A and 2A, *l_pr_*), and 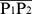 can be obtained from: (i) geometric parameters obtained from the three-dimensional image of the humerus (*R_c_*, *d_e_*, ε, Fig. 2B); (ii) the position of the humeral medial epicondyle at each flexion angle (Fig. 2B), and (iii) the position of the elbow flexion axis, which depends on the carrying angle (λ) and varies throughout the flexion-extension range (see Fig. 1 and Eq. 1) [10]. Then, the distance *l*_1_ in maximum elbow extension can be calculated as follows: 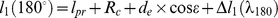



where Δ*l*_1_(λ_180_) is obtained from Eq. 1. Distance 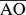 is the curvature of the radius, measured on dry bone [4], [15]. Distance 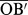 (projection of 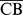 segment on plane P_3_), is dependent on λ value and can be obtained for each position of the elbow using Eq. 2.

Then, 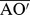 and 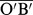 can be respectively calculated from 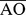 and 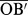 by determining distance 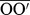 [4]. Knowing the position of point B and its projection on plane P_3_ (Fig. 3), we can calculate, at any flexion angle of the elbow, distance 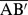, angle β, 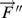 and 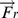 and, therefore, the value of E_rot_.

## Results

**Figure 4:**
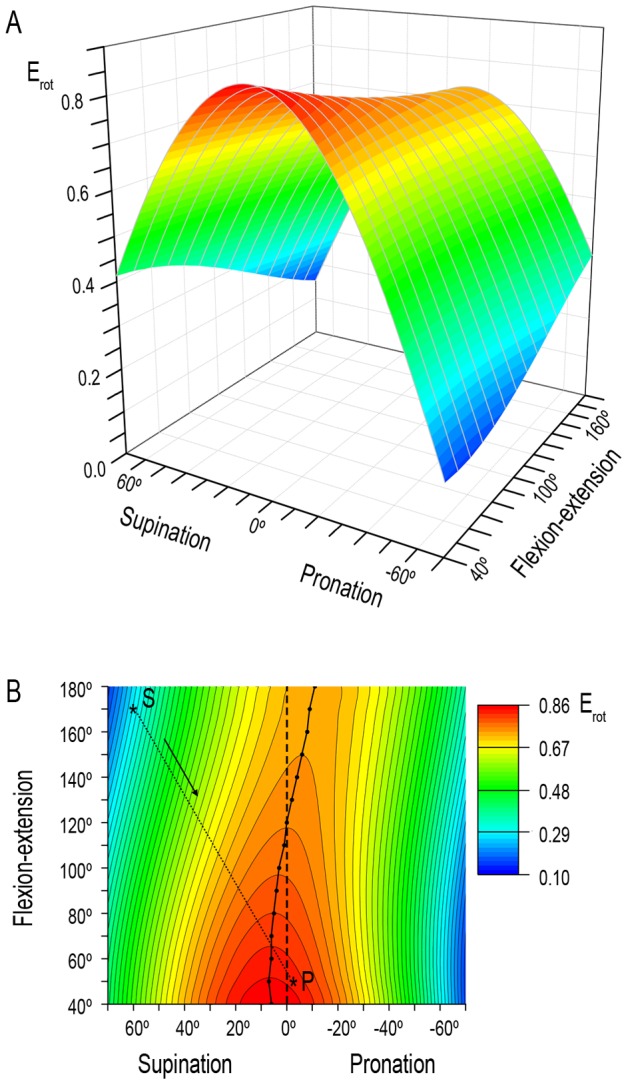
Forearm rotational efficiency (E_rot_) as a function of pronation-supination and flexion-extension angles. **A:** Three-dimensional surface showing efficiency values at each forearm and elbow angles. **B:** Projection of the three-dimensional surface on XY plane showing efficiency ranges at each forearm and elbow angles. The points connected by a continuous line indicate the forearm positions where efficiency is maximal for each elbow angle. The dashed line indicates the neutral position of the forearm. The dotted line shows a trajectory followed by the forearm to reach an object with the elbow almost fully extended (170°) and the forearm supinated (60°) (asterisk S) and to bring it close to the face (50° of elbow flexion and −5° of pronation) (asterisk P). This trajectory, which direction is indicated by the arrow, is the shortest and entails an increase of about 190% in the efficiency value.

Figure 4 shows PT E_rot_ throughout the flexion-extension and the pronation-supination ranges. Rotational efficiency for each elbow position is maximal when the forearm is close to the neutral position (position attained when the thumb points superiorly and the palm faces medially, at 0° of pronation-supination [10]) and is minimal in the extremes of the pronation-supination range. Maximum E_rot_ for each elbow position undergoes a nonlinear growth of 18% from full extension (180°) to full flexion (40°). The position of maximum E_rot_ displaces from −11° of pronation at 180° of elbow extension towards 6° of supination at 40° of elbow flexion (40°) (Fig. 4B). At 120° of elbow flexion, E_rot_ reaches its maximum when the forearm is in the neutral position.

**Figure 5:**
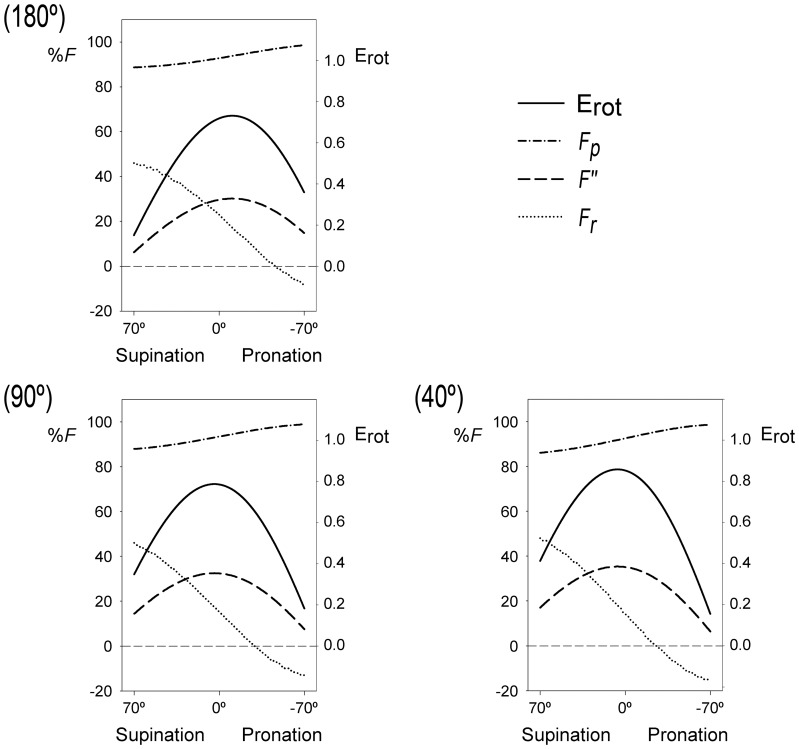
Relative values for 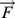 components and forearm rotational efficiency (E_rot_) in three elbow positions. Full extension (180°), intermediate position (90°) and full elbow flexion (40°) scenarios are shown.

The force that PT exerts on the radius is represented by 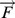 (Fig. 1). The projection of this vector on the axial plane P_3_ is represented by 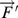 (Fig. 3), whereas the projection on the vertical axis is named 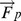 (Fig. 1). 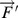 can be decomposed into two vectors: 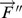, which is tangent to the trajectory of the radial rotational movement, and 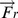, which is directed to the rotation center (Fig. 3). Figure 5 shows the relative values for these components at three elbow angles (40°, 90° and 180°). 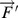 is greater in supination than in pronation, regardless of the elbow angle. The modulus for the perpendicular component of 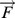 (*F_p_*) displays values that vary between the 86% and 99% of *F* value, depending on the pronation-supination and flexion-extension angles. This component increases as the forearm pronates, regardless of the elbow position. It is also slightly greater in extension than in flexion of the elbow.

Concerning the radial component (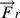), its modulus values are approximately the 50% of *F* value in a full supination position for the entire flexion-extension range. These values decrease as the forearm pronates, reaching negatives values in pronation. The forearm position where *F_r_* = 0 depends on the elbow angle. At 180° of elbow extension, the modulus for 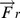 reaches 0 at −46° of pronation, whereas in full elbow flexion this occurs at −25° of pronation. Therefore, this component reaches lower negative values, i.e. higher values for its modulus being the vector negative, as the elbow flexes (up to 15% in full flexion). Regarding 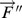, which modulus does not reach the 40% of *F*, its values change in a similar way to the values of E_rot_. The pronation-supination angle where *F″*  =  *F_r_* is dependent on the elbow position. At 180°, it is close to the neutral position of the forearm (at 11° of supination). As the elbow flexes, it displaces towards a more supinated position of the forearm, reaching 31° of supination in full flexion.

**Figure 6:**
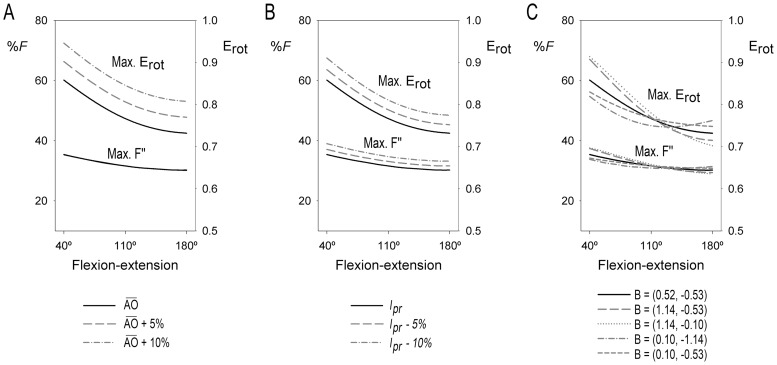
Simulated changes on maximum rotational efficiency (Max. E_rot_) and maximum *F″* value (Max. *F″*). Simulations are shown throughout the flexion-extension range. **A:** Simulations of an increase in 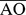. Note that the relative values for *F″* of the three simulations overlap. **B:** Simulations of a decrease in *l_pr_*. The effect caused by an increase in 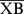 is similar to the effect caused by the decrease in *l_pr_* shown here. **C:** Simulations with different coordinates of point B (cm).

Several simulations were carried out to assess how E_rot_ and 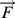 components are modified by changes in some osteometric parameters. From the mathematic expression to calculate E_rot_ (see Materials and Methods and Figures 1, 2 and 3), it is easily inferable that a greater curvature of the radius (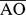), and so a greater rotational radius (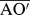), leads to a proportional increase in the E_rot_ values (Fig. 6A). Moreover, any change in one or several osteometrical parameters that causes an increase of α and/or β angles will consequently enhance E_rot_ (see Materials and Methods). Hence, the moment of force can be improved by rising either the tangential force (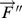) or the curvature of the radius (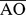). Although both scenarios would cause an increase in E_rot_, they are related to different structural characteristics. A 10% rise of 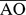 has a minor effect on the components of 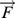, being 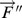 modulus constant (Fig. 6A) and slightly reducing 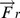 modulus at −70° of pronation, from 8–15% to 6–13% of *F* value, depending on elbow position. When a similar increase in E_rot_ is simulated by a 10% decrease of *l_pr_*, a completely different behavior of 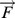 vectors can be observed: 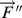 modulus increases proportionally to E_rot_ during the entire flexion range (Fig. 6B), whereas 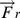 modulus rises from 46–48% to 49–51% of *F* value at 70° of supination and from 8–15% to 10–19% at −70° of pronation. A 10% increase of 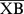 has broadly the same effect on E_rot_ and 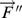 and 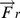 moduli than the reported 10% decrease of *l_pr_*. 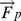 is not meaningfully modified by changes in the abovementioned parameters, whereas the forearm positions where 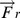 modulus reaches 0, where 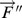 and 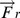 become equal and where E_rot_ is maximal are considerably affected by these changes. All this information is summarized in Table 2.

**Table 2:** Effect of the simulations on 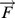 components and rotational efficiency (E_rot_) for three elbow positions (180°, 90° and 40°).

Simulation	Forearm position where *F_r_* = 0			Forearm position where *F″* = *F_r_*			Forearm position where E_rot_ is maximal		
	180°	90°	40°	180°	90°	40°	180°	90°	40°
Original values	−47°	−29°	−25°	11°	28°	31°	−11°	4°	6°
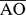 increase (+10%)	−52°	−34°	−30°	8°	25°	28°	−11°	4°	6°
*l_pr_* decrease (−10%)	−44°	−27°	−23°	13°	29°	34°	−11°	3°	5°
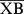 increase (+10%)	−42°	−26°	−23°	14°	29°	33°	−10°	3°	5°

Conversely to the abovementioned simulations, the direction of the change in E_rot_ and 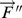 modulus (increase or decrease) caused by a change in the position of point B depends on the elbow angle (Fig. 6C). When coordinate *x* increases, maximal E_rot_ and 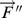 modulus rise in flexion of the elbow and decrease in extension. When this coordinate is lower, the values for these parameters decrease in flexion and rise in extension. When coordinate *y* is closer to 0, E_rot_ and 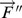 modulus increase in flexion and fall in extension, whereas when this coordinate is lower, these parameters decrease in flexion and rise in extension.

Efficiency is also lower when the carrying angle (λ) is greater. A change of 5° in this angle leads to a variation of about 6.5% of the maximum E_rot_ value at 180° of elbow extension. This effect is lesser as the elbow flexes and it is null in full flexion (40°).

## Discussion

### Rotational efficiency, force components and structural implications

In the current study, PT E_rot_ has been assessed throughout the entire flexion-extension range using three-dimensional technology. The variation of E_rot_ obtained from our innovative biomechanical model is in agreement with the results of kinematic studies using cadaveric specimens [16], [17] and virtual and resin models of the upper-limb skeleton [18]–[20], as well as with analysis on forearm discomfort [21] and on electromyographic signals of the forearm pronators [7].

Pronator teres E_rot_ is dependent on the skeletal structure of the arm, elbow and forearm, which in turn can be modified by the usage of this muscle. In this regard, the analysis of E_rot_ has enabled to study the effect of the components of PT force vector on the upper-limb skeleton. The perpendicular component of this vector (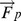) shows high relative values, which indicates that an important part of PT force is employed in a direction parallel to the rotational axis of the forearm, i.e. compressing the radius lengthwise towards the distal epiphysis of the humerus. This compression is also produced during the contraction of other upper-limb muscles, such as biceps brachii and the wrist and fingers flexors. As regards PT, the compressive effect on the radius is higher in pronation than in supination and slightly decreases from elbow extension to maximum flexion. Although the differences are slight, this indicates that pronated positions of the forearm enhance the curvature of the radius through radial compression in a greater degree than supinated positions. This effect is independent of the skeletal structure, as 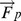 is broadly unaffected by changes in the parameters used to calculate E_rot_.

When the forearm is pronated, biceps brachii is reflexly inhibited and plays little if any role in flexion, because it would supinate the forearm during contraction [22]–[24]. As mentioned, the perpendicular component of PT force is greater on the prone forearm. This increase may partially compensate the lack of action of biceps brachiii, enhancing the assisting role of PT as elbow flexor and joint stabilizer in this position.

The component 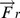, which directs to the rotation center, reaches negative values in forearm pronation. When this component is negative, the vector directs opposite to the rotational center, and so it enhances the curvature of the radius. Negative values are reached in a position of the forearm that gets closer to the neutral position as the elbow flexes. Therefore, the curvature of the radius (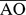) is enhanced during pronation in flexion rather than in extension of the elbow. In any case, the relative values for 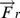 responsible for the curvature of the radius are low when compared to values for 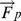, which suggests that radii with marked curvatures are more probably associated to compression forces from PT, among other muscles, than to forces applied perpendicularly to the forearm axis. These findings are in agreement with a previous empirical study that revealed that the pattern of muscular loading exerted on the apex of the radial shaft curvature by the PT muscle plays an important role as a mechanical stimulus involved in diaphyseal bowing [15].

The effect that the skeletal structure and form have on E_rot_ and on the force vectors has also been assessed. The results show that a greater bowing of the radius entails an increase of E_rot_. This is consistent with previous studies suggesting an enhancement of PT action and forearm rotational power by a markedly bowed radius [3], [25]–[28]. An increase of the radial curvature also causes that 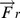 becomes negative in a more pronated position of the forearm, and so its modulus reach lower values in full pronation. Therefore, the radius is more easily bowed when its curvature is low.

The radial location of PT muscle also affects E_rot_: at any elbow angle, E_rot_ increases when this enthesis is more proximally located, which is consistent with previous observations in full elbow extension [3]. Moreover, radii with a more proximal enthesis for PT muscle lead to the reach of negative values of 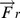 closer to the neutral position of the forearm. Therefore, these radii entail lower negative values for this component, i.e. higher values for its modulus being the vector negative, which stimulates radial curvature.

Concerning the humeral medial epicondyle, E_rot_ also tends to increase in all elbow positions as this structure enlarges. Even though the current analysis uses a different approach to quantify humeral medial epincondylar projection (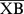), the results are consistent with previous studies [3], [5], [29]. Moreover, an enlargement of the medial epicondyle has the same effect on 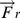 than a more proximally located radial enthesis for PT, and therefore a more medially projected epicondyle enhances radial curvature.

The orientation of the medial epicondyle is also relevant for the determination of E_rot_. The alteration of this orientation causes changes in 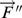 that lead to changes in E_rot_. A more posteriorly oriented epicondyle, i.e. more retroflexed [5], [30], is associated with a greater E_rot_ in full extension of the elbow, whereas an epicondyle with a lower degree of retroflexion has greater values of E_rot_ in full flexion. Moreover, a more proximally oriented epicondyle enhances E_rot_ in full flexion, whereas when it is more distally oriented, E_rot_ increases in extension. The orientation of the medial epicondyle was reported to present a partially activity-dependent plasticity, and so it was hypothesized that it can be modified to enhance certain abilities [30]. In this regard, and in agreement with the abovementioned relationship between E_rot_ and epicondylar orientation, a simple observation of the upper-limb positioning shows that a habitual and continued contraction of PT in full elbow flexion would reorient the epicondyle towards a more proximal position. Conversely, this reorientation would occur distally in full elbow extension. The reported association between the orientation of the medial epicondyle and E_rot_ may have important implications on the evolutionary pathway of the upper-limb skeleton, as this structure plays a central role in the locomotor diversity of primates [5], [31]–[33].

Regarding the carrying angle of the elbow [11], this is the first quantitative insight into its biomechanical implications on forearm rotation. An increase in this angle leads to lower values of E_rot_. This effect is the greatest in full elbow extension and becomes lower as it is flexed, given that carrying angle is maximal in full extension, decreases during flexion and reaches 0° when the elbow is completely flexed [10], [12]–[14].

### Functional implications of forearm rotation

The biomechanical model described here enables to make a thorough quantitative approach to clarify some mechanical aspects about the relationship between PT and forearm and elbow motion. For instance, the results indicate that maximal E_rot_ for each elbow angle is close to the neutral position of the forearm. This position has been commonly associated to the functional position, which minimizes the expenditure of muscular energy as it implies a natural equilibrium between antagonist muscles [10]. Moreover, it entails an enhancement of the precision of the grip, because the forearm axis is in line with the pronation-supination axis [10], [34].

Although skeletal structures with different morphologies can have the same E_rot_, they do not have to imply the same abilities in terms of precision. For instance, if the same force is exerted by PT on two upper-limbs with the same E_rot_, the one with the greatest curvature will be able to reach a lower rotational angle (File S1 and Fig. S1), and thus a greater rotational precision.

The stability of the elbow joint has relevant implications for sports medicine and other pathological conditions [14], [35], [36]. During pronation, 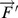 plays an important role in the stability of the radio-ulnar joint (see File S1 and Fig. 3), whereas 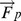 participates in the radio-humeral stability (see Fig. 1A). The global stability of the elbow is thus enhanced by the combination of both components. Although 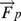 is slightly lower when the forearm is supinated, the great 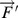 value in this position leads to a better fitting between the radius and the ulna than in pronation [37].

Rotational efficiency and the forces that act during pronation may be associated to the estimations of the discomfort levels done by Mukhopadhyay et al. [38], as the movements implying a high level of discomfort correspond to positions where E_rot_ is low. Moreover, the concept “efficiency” provides information about the rotational stability of a given position, as the work needed to modify the forearm rotational angle depends directly on the value of E_rot_ in each position (see File S1). For a given pronation movement, the model can be used to determine the trajectories of the hand that entail the minimal expenditure of energy for PT. In humans, pronation is usually performed when an object has been reached with the hand supinated and the elbow extended and is then brought closer to the body. The final position of this movement will entail a full flexion of the elbow and a close-to-neutral position of the forearm. This movement implies an increase of PT E_rot_, which indicates that the energy expenditure of this muscle diminishes when the elbow is flexed (see Fig. 4B). The results show that a great part of this trajectory entails upper-limb positions where 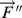 and 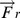 have a similar value, i.e. around the forearm position where *F″*  =  *F_r_*. This indicates that during this trajectory the forces that the elbow joint is submitted to are quite equilibrated.

These two conditions (equilibrium between forces and increase of E_rot_) are not observed for other trajectories, such as those where the end point is close to full pronation with the elbow flexed. In this case, the final part of the trajectory entails a decrease of E_rot_ and very different values of these two force components. If the forearm was very regularly used to perform this movement with important mechanical loads, the disequilibrium could have pathological consequences, as a result of heavy tensions in the radio-ulnar proximal joint. In order to minimize the expenditure of energy and to rebalance the forces, the radial curvature could increase. As reported by our simulations, a rise of the curvature of the radius causes an increase of E_rot_ and a displacement of the position where *F_r_* = 0 towards full pronation, as well as a displacement of the position where 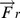 and 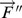 become equal from supination towards a position closer to the neutral.

Therefore, the results of the current study also indicate that the upper-limb skeleton may experience plastic changes as a result of PT activity when it is used in positions where E_rot_ is low and forces are not equilibrated. These changes entail an improvement of these parameters, in order to adjust to the unfavorable conditions. Conversely, if the upper-limb is mainly used in positions with high E_rot_ values, PT will not trigger changes in the skeletal structure. Overall, E_rot_ and its relationship with energy expenditure and rotational stability, as well as the relative values of the force components, which are linked to the joint stability, provide a global insight into pronation and its association to the forearm skeletal structure. These findings will be useful for future analyses on ergonomics and orthopaedics of the upper-limb skeleton, as they provide relevant information about the relationship between PT functionality and the skeletal structure.

Applying this biomechanical model on studies about comparative anatomy of primate taxa would provide valuable information about the functional meaning of the upper-limb skeletal interspecific differences, which will probably be associated to the locomotor abilities of each taxon. Moreover, an analysis of PT E_rot_ and force components on a fossil primate would very useful for inferring its upper-limb involvement in locomotion.

Some skeletal parameters essential for the determination of PT functionality, such as the radial curvature and the medial epicondylar form, are reported to be plastic and related to the usage of PT muscle [15], [30]. Therefore, this biomechanical model would also be of great interest if applied to ancient human populations. Differences in parameters derived or related to E_rot_ between sexes, groups or populations would account for differences in occupational and habitual activities.

## Supporting Information

Figure S1
**Diagrams showing the displacement caused by a force in different scenarios.** **A:** Linear displacement (Δs) and rotational angle (Δθ) caused by a force 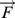 on a rotating body, being the center of rotation O and the rotational radius R. **B:** Representation of the differences in the force (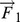 and 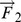) that are needed to obtain the same rotational angle when the rotation performed by pronator teres muscle starts at a different point (A_1_ and A_2_) (see Fig. 3). **C:** Representation of the differences in the rotational angle (Δθ_1_ and Δθ_2_) obtained when the same force (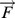) is applied by pronator teres muscle on two radii with different rotational radius (r_1_ and r_2_) (see Fig. 3). Note that the same linear displacement (Δs) is obtained in both cases.(TIF)

File S1 **Rotating work during forearm pronation, stability and muscular energy expenditure.**
(DOCX)
