# Supplementary material for: Biomechanics of Forearm Rotation: Force and Efficiency of Pronator Teres
Source: PLoS One. 2014 Feb 28;9(2):e90319. doi: 10.1371/journal.pone.0090319 (PMC3938685; doi:10.1371/journal.pone.0090319)
Supplement: File S1 — Rotating work during forearm pronation, stability and muscular energy expenditure. (DOCX) [file pone.0090319.s002.docx]

**Rotating work during forearm pronation, stability and muscular energy expenditure**

*Pronation work as a function of forearm rotational angle*

A force $\overrightarrow{F}$ acting on a rotating body, shown in Figure S1A, produces an angle of rotation (Δθ). The work done by this force is:

$$\Delta\text{W}=F\times\Delta\text{s}=F\times\text{R}\times\Delta\text{θ}$$

The product $F\times\text{R}$ defines the torque of $\overrightarrow{F}$ about the rotational center O:

$$\Delta\text{W}=\text{M}\times\Delta\text{θ}$$

By using these formulae in our model of forearm rotation performed by PT (see Figs. 3 and S1B) at two different points (1 and 2), we obtain:

$\Delta\text{W}_{1}=F_{1}\times\text{r}_{\text{rot}}\times\Delta\text{θ}=\text{M}_{1} \times\Delta\text{θ}$ (close to maximum supination)

$\Delta\text{W}_{2}=F_{2}\times\text{r}_{\text{rot}}\times\Delta\text{θ}=\text{M}_{2}\times\Delta\text{θ}$ (close to neutral position)

And for a given PT muscular force ():

$$\Delta\text{W}_{1}=F_{m}\times\text{cosα}_{1}\times\text{cosβ}_{1}\times\text{r}_{\text{rot}}\times\Delta\text{θ}=F_{m}\times\text{E}_{\text{rot1}}\times\Delta\text{θ}$$

$$\Delta\text{W}_{2}=F_{m}\times\text{cosα}_{2}\times\text{cosβ}_{2}\times\text{r}_{\text{rot}}\times\Delta\text{θ}=F_{m}\times\text{E}_{\text{rot2}}\times\Delta\text{θ}$$

The following equation can be used to assess the relationship between the works produced by PT to rotate the radius an angle of θ degrees in two positions with different E_rot_ values:

$$\Delta\text{W}_{2}= \Delta\text{W}_{1}\times\text{E}_{\text{rot2}}/\text{E}_{\text{rot1}}$$

For example, at 90º degrees elbow flexion, point 1 (Fig. S1B) is at 60º of supination (E_rot_ = 0.35) and point 2 is at 5º of supination (E_rot_ = 0.77):

$$\Delta\text{W}_{2}/\Delta\text{W}_{1}=\text{E}_{\text{rot2}}/\text{E}_{\text{rot1}}=2.2$$

This means that the work necessary to rotate the radius θ degrees is 2.2 times greater if the radius is in position 2 than if it is in position 1. Conversely, the energy expenditure of PT to stay in position 2 would be 2.2 times lower than in position 1.

This approach based on the expenditure of energy clarifies two issues: (i) for a given force, PT expends less energy when the forearm is in a position where E_rot_ is maximal than in any other position, and (ii) the maximal rotatory capacity that a specific PT muscle can get will occur in a position of maximum E_rot_.

*Pronation work in upper-limbs with different structural characteristics*

The simulations performed using the model show that similar or the same E_rot_ is obtained in upper-limbs with different structural characteristics, e.g. decreasing *l_pr_* value or increasing radial curvature. In order to obtain the same lineal displacement (Δs) by applying the same muscular force on two upper-limbs with the same E_rot_ but with different radial curvatures or rotational radius (Fig. S1C):

$$\Delta\text{W}=F\times\Delta\text{s}=F\times\text{r}_{1}\times\Delta\text{θ}_{1}=F\times\text{r}_{2}\times\Delta\text{θ}_{2}$$

That is:

$$\text{r}_{1}/\text{r}_{2}=\Delta\text{θ}_{2}/\Delta\text{θ}_{1}$$

A greater rotational radius implies a lower rotational angle. Conversely, in order to obtain a specific angle θ, the tangential force that has to be applied is greater as the radius is more curved.
